# A Comprehensive Study of the Relationship between the Production of *β*-Lactamase Enzymes and Iron/Siderophore Uptake Regulatory Genes in Clinical Isolates of *Acinetobacter baumannii*

**DOI:** 10.1155/2021/5565537

**Published:** 2021-03-17

**Authors:** Mahyar Porbaran, Hamed Tahmasebi, MohammadReza Arabestani

**Affiliations:** ^1^Department of Microbiology, Faculty of Basic Sciences, Islamic Azad University, Hamedan Branch, Hamedan, Iran; ^2^School of Medicine, Shahroud University of Medical Sciences, Shahroud, Iran; ^3^Microbiology Department, Faculty of Medicine, Hamadan University of Medical Sciences, Hamadan, Iran

## Abstract

**Background:**

The iron/siderophore uptake system (IUS) involved in the *Acinetobacter baumannii* pathogenicity. However, IUS's role in antibiotic resistance and the production of *β*-lactamase enzymes of *A. baumannii* are unclear. This study aimed to investigate the relationship between the production of *β*-lactamase enzymes and IUS regulatory genes in clinical isolates of *A. baumannii*. *Methods. A. baumannii* isolates were collected from clinical isolates using biochemical tests. The antibiotic resistance patterns and *β*-lactamase-producing strains were identified using the disk diffusion method (DDM). Also, IUS genes were detected by the polymerase chain reaction (PCR) method.

**Results:**

Seventy-two (72) *A. baumannii* isolates were collected from a different clinical specimen. Gentamicin-resistant strains (43%) had the highest frequency, and aztreonam-resistant strains (12.5%) had the lowest frequency. Also, the distribution of AmpC and MBL producing isolates were 27.7% and 35%, respectively. Moreover, the frequencies of *basD*, *bauA*, *pld*, *paaE*, *entA*, *feoB*, *hemO*, and *tonB* genes were as follows: 12.5%, 15.2%, 11.1%, 15.2%, 19.4%, 16.6%, 23.6%, and 6.9%. Further, a strong correlation was observed between the abundance of *β*-lactamase-producing strains and IUS genes.

**Conclusions:**

Based on our knowledge from this study, the association between *β*-lactamase production and IUS genes in *A. baumannii* plays an essential role in the emergence of drug-resistant strains.

## 1. Introduction

*Acinetobacter baumannii* (*A. baumannii*) has become an increasingly important human pathogen connected with infections acquired in hospitals [1]. It has become more challenging to treat *A. baumannii* infections in the hospitalize portions because of their resistance to significant groups of antimicrobial agents such as *β*-lactams [2]. Various resistance mechanisms make *A. baumannii* a successful pathogen of the 21^st^ century [3]. Extended-spectrum *β*-lactamases (ESBL) belong to the class A *β*-lactamases. Metallo *β*-lactamases (MBL), which have metal ions governing the active site, belong to the class B *β*-lactamases [4–6]. Class C *β*-lactamases are acinetobacter-derived cephalosporinases or the chromosomally encoded AmpC enzymes. Also, in class *D β*-lactamases, oxacillinases are involved in resistance to carbapenems. However, there is now a crucial need for new antibacterial strategies due to the spread of pathogen populations [7–9].

In *A. baumannii,* virulence factors and biofilm formation play a crucial role in disease progression in an infected host [10–12]. Another virulence factor studied in detail is *A. baumannii* to survive in an iron-limited environment, like the human host [13]. In response to low iron, *A. baumannii* produces the siderophore acinetobactin to acquire this essential micronutrient. A better understanding of the acinetobactin system could lead to new antimicrobial drugs to treat infections caused by multidrug-resistant (MDR) *A. baumannii* infections [10]. However, *A. baumannii* synthesizes siderophores which are relatively low-molecular-weight agents capable of converting polymeric ferric oxy-hydroxides to soluble iron chelates under low iron stress. The ability of bacteria to assimilate iron is related to invasiveness [3, 14].

However, over the past decade, a need for new antimicrobial compounds has arisen with the advent of MDR bacteria, such as *A. baumannii*, plaguing our healthcare systems [13, 15]. Since iron is essential for pathogenic bacteria to cause a successful infection, siderophore-mediated iron acquisition systems have been exploited as potential therapeutic targets [13]. One strategy utilizes the siderophore uptake machinery to deliver an antibiotic conjugated to the siderophore [2]. Nevertheless, in this study, to determine the relationship between *β*-lactamase enzymes and iron uptake pathways, we investigated the iron uptake system's role in the emergence of resistant strains.

## 2. Materials and Methods

### 2.1. Design of Study and Sample Collections

A cross-sectional study was performed on patients admitted to Hamedan's Hospital, attached to Iran's Hamedan Medical School. The study was conducted between November 2018 and April 2020. Six hundred ninety clinical samples were collected, including wound swab, urine, blood, ascetic fluid, endotracheal fluid, bronchoalveolar lavage—inclusion criteria for patients presenting with signs of bacterial infection from bacterial culture examination. However, patients without bacterial infection symptoms, incomplete medical record data, or those who underwent nonstandard bacterial culture screening procedures were excluded from the study. After considering the inclusion and exclusion criteria, samples are used for culture and further biochemical investigations. Samples are inoculated in blood agar, MacConkey agar, and nutrient agar.

### 2.2. Antibiotic Susceptibility Test (AST)

The AST was detected by disk diffusion methods (DDM). Twelve antibiotic discs (trimethoprim/sulfamethoxazole, piperacillin/tazobactam, amikacin, ciprofloxacin, ofloxacin, gentamicin, cefoxitin, imipenem, cotrimoxazole, cefepime, ertapenem, and aztreonam) all owned by Mast, UK, were used to evaluate the AST. Interpretation of antimicrobial sensitive or resistance zone was done by CLSI 2019 Guidelines [16].

### 2.3. Detection of AmpC and MBL Producing Strains

The AmpC and MBL producing *A. baumannii* were detected by the DDM [7].

### 2.4. Detection of Biofilm-Forming Strains

Microtiter dish assay was used to identify the biofilm-forming *A. baumannii* [17].

### 2.5. Total DNA Extraction

Genomic DNA was extracted using the method of Tahmasebi et al. [7]. By loading of 5 *μ*l of the extracted DNA in 1% agarose gel, the extracted quality was checked.

### *2.6. Determination of Iron/Siderophore Mediate* Gene

According to Abdi et al. [3], the PCR method was used for the detection of the IUS gene. For partial gene, the PCR conditions used to amplify the iron/siderophore mediate gene were the same as described in Table 1. Moreover, PCR was performed using a C1001 Bio-Rad thermal cycler. Finally, 5 *μ*l of amplified product is electrophoresed on 1.5% agarose gel at a constant of 80 V for 60 min [3].

### 2.7. Statistical Analysis

In this study, GraphPad Prism software 8 (GraphPad Software, Inc., San Diego, CA) was used to analyze the data. Chi-square, two-way ANOVA, Tokay, and *t*-test were also used to examine the relationship between different variables. A *p* value of 0.05, 0.01, and 0.001 was considered as the level of significance.

## 3. Results

Out of 690 isolates of different clinical isolates, 72 isolates of *A. baumannii* were collected. According to Table 2, most samples were obtained from wound 23 samples (31.9%), followed by urine 21 samples (29.1%), blood 18 (25%), and body fluids 10 (13.8%).

### 3.1. Antibiotic Suspectable Profile

Table 2 shows the percentage of sensitivity, intermediate, and resistance of the tested *A. baumannii* isolates. More than 30% of the isolates were resistant to gentamycin (43.0%), ofloxacin (30.5%), and ciprofloxacin (40.2%). Only nine isolates (12.5%) were resistant to aztreonam. Further, 22 isolates (30.5%) and 13 isolates (18.0%) were considered MDR and XDR, respectively.

### 3.2. Prevalence of MBL and AmpC Producing Strains

**Figure 1:**
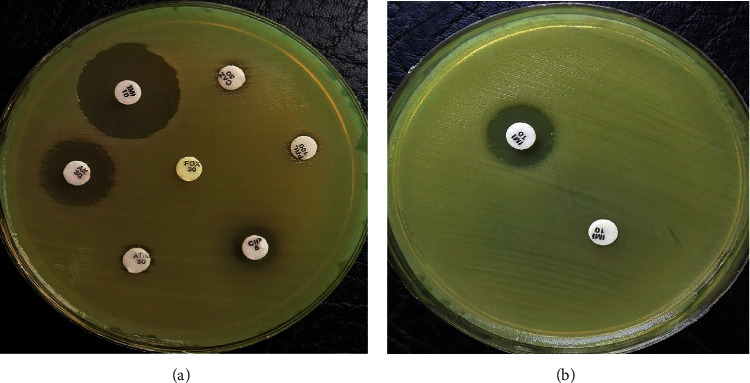
Evaluations of antibiotic resistance pattern and AmpC and MBL producing strains by disk diffusion method. (a) Resistant to cefoxitin (FOX) disk indicates AmpC producing strain. (b) The MBL producing isolates were considered positive when the zone diameter difference between imipenem + EDTA and imipenem discs was more extensive than 7 mm.

Table 2 and Figure 1 show that 27.7% and 36.1% of *A. baumannii* isolates were AmpC and MBL producers, respectively. Also, coexitance of AmpC and MBL enzymes was observed in 12 isolates (16.6%).

### 3.3. Prevalence of Biofilm-Forming A. baumannii

The frequency of biofilm-producing *A. baumannii* is shown in Table 2. Among the 72 isolates of *A. baumannii*, 39 biofilm-forming isolates (54.1%) and 33 nonbiofilm-forming isolates (45.8%) were detected.

### 3.4. Prevalence of Iron/Siderophore Mediate Gene

**Figure 2:**
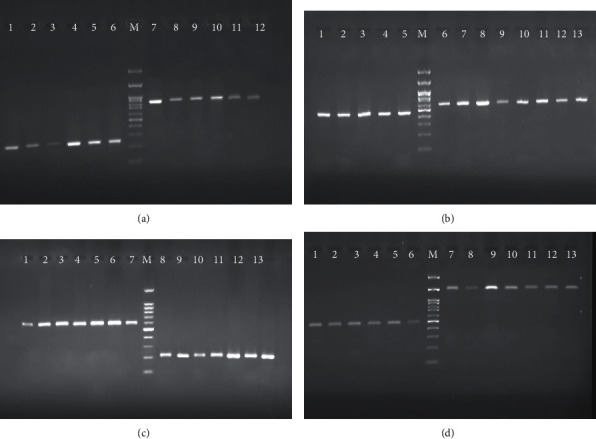
The amplification result of iron/siderophore mediate gene in *A. baumannii*. (a) *basD* gene with 868bp and *bauA* gene with 224bp; well 1: positive control; wells 2 to 7: positive strains with *basD* gene; well 11: positive control; wells 8 to 10: positive strains with *bauA* gene. (b) *pld* gene with 662bp and *paaE* gene with 593p; well 1: positive control; well 3 to 10: positive strains with *paaE*; well 13: positive control; wells 7 to 12: positive strains with *pld*. (c) *feoB* gene with 636bp and *hemO* gene with 215bp; wells 1: positive control; wells 2 to 5: positive strains with *hemO*; wells 10: positive control; wells 6 to 9: positive strains with *pld*. (d) *entA* gene with 504bp; wells 1: positive control; wells 2 to 9: positive strains with *tonB* gene. (M) Ladder 100bp.

The distribution of siderophore genes is illustrated in Table 2 and Figure 2. Among 72 isolates of *A. baumannii*, 9 isolates (12.5%) carry *BasD* gene, 11 isolates (15.2%) carry *BauA* and *paaE* genes, 8 isolates (11.1%) carry *Pld* gene, 12 isolates (16.6%) carry *feo B* and *entA* genes, 17 isolates (23.6%) carry *hemO* gene, and 5 isolates (6.9%) carry *tonB* gene.

### 3.5. Statistical Analysis

**Figure 3:**
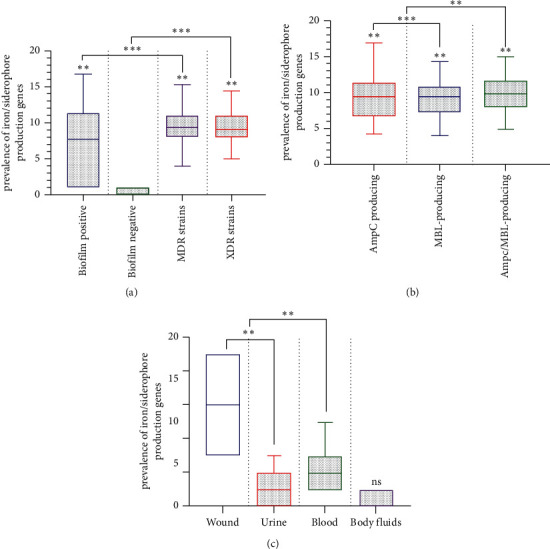
Correlation between the iron/siderophore mediate gene, biofilm formation, separated clinical isolates, and antibiotic resistance in *A. baumannii*. (a) Correlation between antibiotic resistance, biofilm formation, and iron/siderophore mediate gene. (b) Correlation between *β*-lactamase enzymes and iron/siderophore mediate gene. (c) Correlation between clinical sample type and iron/siderophore mediate gene. Each data set was analyzed using the Student's *t*-test, Tokay, and the two-way ANOVA and was presented as mean + SEM. ^*∗*^*p* value <0.05; ^*∗∗*^*p* value <0.01; ^*∗∗∗*^*p* value <0.001; ^*∗∗∗∗*^*p* value <0.0001. ns: nonsense.

Based on Figure 3, a statistical association was detected when the IUS had compared virulence factors. Using two-way ANOVA tests, *t*-test, and Tukey, IUS genes' high frequency was significantly reported in AmpC/MBL producing isolates. However, biofilm formation is positively associated with antibiotic resistance, siderophore, and *β*-lactamase productions (*p* < 0.05). In terms of MDR and XDR strains, harboring IUS genes and biofilm formation was strongly associated with *β*-lactamase production. In contrast, AmpC/MBL *β*-lactamase production was positively associated with siderophore production (*p* < 0.001).

## 4. Discussions

Acinetobacter's hospital infection has developed as a serious threat to the healthcare system due to the emergence of pan resistance from multiresistance. In recent years, *A. baumannii* has become a worldwide concern due to severe nosocomial infection.

In the current study, *A. baumannii* isolates obtained from several clinical samples were collected. Most samples were obtained from wound samples (31.9%) followed by urine (29.1%), blood (25.0%), and body fluids (13.8), which is concordance with study Lukovic et al. and Sheikh et al., where there are maximum isolates wound samples [18, 19].

The current study revealed the lowest frequency of resistance to aztreonam (12.5%), followed by TMP/SMX (23%) and PIP/TAZ (25%). This finding was also reported by Armin et al. and Kamali et al. [20, 21]. In line with Jasemi et al.'s [22] study, 27.7% and 36.1% of isolates were considered XDR and MDR, respectively. However, these findings were in contrast to the data reported from India, Sweden, and Italy [4, 23, 24]. Also, 27.7% and 36.1% of *A. baumannii* produced AmpC and MBL enzymes, respectively. A similar pattern of results was obtained in El-Bakyet al. and Kaur and Singh. They showed that about 35% of *A. baumannii* isolates produced AmpC and MBL enzymes [8, 25]. However, these findings contrast with the previous studies in India and Pakistan [26, 27]. Also, by the time *A. baumannii* isolates acquire resistance to *β*-lactams and carbapenems, they often acquire resistance to several other antimicrobial agents as well [26].

According to the present study, the frequencies of *basD, bauA, pld, paaE, feoB, hemO, tonB*, and *entA* genes in *A. baumannii* clinical isolates were 12.5%, 15.2%, 11.1%, 15.2%, 16.6%, 23.6%, 6.9%, and 19.4%, respectively. However, this frequency has not previously been described. The levels observed in this investigation are far below those observed by Abdi et al. [3]. They reported that frequencies of *tonB*, *barA*, *feoB*, *entA*, and *hemO* genes were 85%, 97%, 99%, 99%, and 95%, respectively. A study conducted by Liu et al. [2] also showed a high prevalence of iron/siderophore genes in *A. baumannii* isolates. Unfortunately, few studies have surveyed the abundance of iron/siderophore mediate genes.

A significant relationship was observed between antibiotic resistance and IUS genes' distribution based on our study (*p* < 0.05). However, there are very few nationwide studies conducted on IUS genes associated with antibiotic resistance and biofilm formation in Iran. Furthermore, we found that AmpC/MBL producing among *A. baumannii* isolates is significantly correlated with increased biofilm formation (*p* < 0.001). A correlation between IUS and antibiotic resistance patterns was also reported by Zeighami et al. Moreover, these authors reported a correlation between IUS and upregulation of the *β*-lactamase gene encoding the AmpC and MBL enzymes. They suggested that IUS played an essential role in the prevalence of antibiotic resistance in clinical isolates of *A. baumannii* [28]. Moreover, following the present results, Kröger et al. and Mohajeri et al. have demonstrated that iron uptake through siderophores' production is another factor of virulence in this organism. However, this finding broadly supports the work of other studies in this area linking iron/siderophore mediate with drug resistance [13, 28, 29].

*A. baumannii* isolates by several mechanisms of resistance can also deferentially express various virulence factors. These factors include siderophore production, biofilm formation, and hemolysis on blood agar [13, 30]. However, the association between virulence and antimicrobial resistance seems to be a highly complex one that is still unclear. Notably, the effect of harboring *β*-lactamase on virulence in *A. baumannii* is not well defined.

Finally, an association between IUS and biofilm formation on the one hand and biofilm formation and antibiotic resistance on the other was determined. The relationship between *β*-lactamase production and biofilm formation has been previously reported among MDR *A. baumannii* isolates [12, 28, 31]. Moreover, the association between motility and siderophore production is not surprising since the former factor was associated with biofilm production. Simultaneously, the latter allows for iron acquisition that is crucial for biofilm formation [14]. These associations reveal a highly complex interplay between the different virulence determinants in *A. baumannii*, especially multifactorial. Therefore, considering this relationship, drug-resistant strains can be regarded as more prone to invasion. On the other hand, *A. baumannii* reduces its sensitivity to antibiotics by using iron/siderophore pathway regulatory systems.

In the present study, IUS pathways in antibiotic resistance in clinical isolates of *A. baumannii* were identified. We demonstrated that iron/siderophore uptake plays an essential role in the prevalence of AmpC/MBL producing strains among *A. baumannii* isolated from clinical specimens. Besides, the presence of *feoB*, *entA,* and *hemO* genes was associated with increased virulence and antibiotic resistance compared to *pld,* and *tonB* was positively associated with AmpC/MBLand siderophore production. Nevertheless, our results confirmed that IUS pathways significantly affect the forming extracellular layers in *A. baumannii*. Thus, suppression of *feoB*, *entA,* and *hemO* genes is one of the best ways to control virulent strains. It also inhibits the iron/siderophore uptake pathway increased biofilm-forming strains' sensitivity to the *β*-lactams antibiotics. Further, these associations could help better understand the complex interactions between antimicrobial resistance and virulence.

## Figures and Tables

**Table 1:** Oligonucleotide sequences used in this study and thermal cycling conditions.

Gene	Sequence of primers	Thermal cycles	Product size (bp)	Ref.
*basD*	F: CTCTTGCATGGCAACACCAC	95°C/5 min; (95°C/1 min, 57°C/60 sec, 72°C/45 sec) X30; 72°C/5 min	868	[2]
	R: CCAACGAGACCGCTTATGGT			
*bauA*	F: TGGCAAGGTGAAAATGCACG	95°C/5 min; (95°C/1 min, 58°C/45 sec, 72°C/45 sec) X35; 72°C/5 min	224	[2]
	R: GCCGCATATGCCATCAACTG			
*pld*	F: CCGTCAATTACGCCAAGCTG	95°C/5 min; (95°C/1 min, 57°C/1 min, 72°C/45 sec) X30; 72°C/5 min	662	[2]
	R: CTGACGCTACCTGACGGTTT			
*paaE*	F: CTATTTAGGCGTTGCTGCGG	95°C/5 min; (95°C/1 min, 59°C/45 sec, 72°C/1 min) X30; 72°C/5 min	593	[2]
	R: CCTTACAACGACAGGTCGCA			
*feoB*	F: AAGTCGCCAACTATGCCGGTGT	95°C/10 min; (95°C/1 min, 57°C/30 sec, 72°C/1 min) X30; 72°C/10 min	636	[3]
	R: AAGGCGCTGCCCATGCAAAAAC			
*hemO*	F: TCGTGGCCGCTCAAAACAAGCA	95°C/5 min; (95°C/1 min, 59°C/1 min, 72°C/1 min) X30; 72°C/5 min	249	[3]
	R: AGGCCGCTAAATTACGTGCAGC			
*tonB*	F: TTGTGGTGCCTCTGCAATCGGT	95°C/5 min; (95°C/1 min, 55°C/1 min, 72°C/1 min) X30; 72°C/5 min	1279	[3]
	R: TCGTGTACCCAAACGAGCAGGA			

**Table 2:** The biofilm-forming capacity of *A. baumannii* and percentages of their iron/siderophore mediate gene.

Biofilm	Antibiotic resistance profiles (NO) (%)																	Total
	IMI	ERT	PIP	GEN	CIP	AMK	FOX	AZT	OFL	CEP	TMP/SMX		PIP/TAZ	MDR	XDR	AmpC	MBL	
Strong	9	11	11	11	11	11	11	9	11	11	11		11	11	11	11	11	11
	47.3	52.3	44.4	35.4	50	52.3	55.0	100	37.9	55.0	64.7		100	50	84.6	55.0	42.3	15.2
Moderate	9	10	7	20	10	10	9	0	16	9	6		6	10	2	9	13	23
	47.3	47.6	38.8	64.5	45.4	47.6	45		55.1	45.0	35.2		33.3	45.5	18.1	45.0	50.0	31.9
Weak	1	0	0	0	0	0	0	0	1	0	0		1	1	0	0	0	5
	5.2								3.4				5.5	4.5				6.9
Nonbiofilm	0	0	0	0	1	0	0	0	1	0	0		0	0	0	0	2	33
	19	21	18	31	22	21	20	9	29	20	17		18	22	13	20	26	72
Total	26.3	29.1	25.0	43.0	30.5	29.1	27.7	12.5	40.2	27.7	23.6		25.0	30.5	18.0	27.7	36.1	

*Iron/siderophore mediate gene*																		
*BasD*	9	9	8	9	9	9	9	9	9	9	9	9		9	9	9	9	9
	47.3	42.8	50.0	3.2	41.0	42.8	45.0	100	31.0	45.0	52.9	81.1		40.9	69.2	45.0	34.6	12.5
*BauA*	11	11	11	11	11	11	11	9	11	11	11	11		11	11	9	9	11
	57.8	52.3	61.1	35.4	50.0	52.3	55.0	100	37.9	55.0	64.7	61.1		50.0	84.6	27.7	34.6	15.2
*Pld*	8	8	8	8	8	8	8	8	8	8	8	8		8	8	8	7	8
	42.1	38.0	44.4	25.8	36.6	38.0	40.0	88.9	27.5	40	47.0	44.4		36.3	61.5	40.0	26.9	11.1
*paaE*	7	11	11	11	9	11	11	9	11	11	11	11		10	9	11	11	11
	36.8	52.3	61.1	35.4	40.0	52.3	55.0	100	31.0	55.0	64.7	61.1		45.5	69.2	55.0	42.3	15.2
*feoB*	9	11	11	12	12	11	12	9	11	12	11	12		11	12	11	9	12
	47.3	52.3	52.3	38.7	57.1	52.3	60.0	100	37.9	60.0	64.7	66.6		50.0	92.3	55.0	34.6	16.6
*entA*	14	13	14	12	12	14	12	9	14	13	14	14		13	13	14	13	14
	73.6	61.9	77.7	38.7	38.7	52.3	66.6	100	48.2	65.0	82.3	77.7		59.0	100	70.0	50.0	19.4
*hemO*	17	15	17	17	17	16	14	9	17	17	17	17		17	12	14	11	17
	89.4	71.4	100	54.8	77.2	76.1	70.0	100	58.6	85.0	100	94.4		77.2	92.3	70.0	42.3	23.6
*tonB*	5	5	5	4	5	5	3	5	5	0	0	4		5	5	5	4	5
	26.3	23.8	27.7	12.9	22.7	23.8	15	55.5	17.2			22.2		22.7	38.4	25.0	15.3	6.9

*Clinical samples*																		
Wound	19	20	10	9	2	11	9	7	16	10	10	9		13	7	9	13	23
	100	95.2	55.5	29.0	9.0	52.3	45.0	77.7	55.1	50.0	58.8	50.0		59.0	53.8	45.0	50.0	31.9
Urine	0	0	2	20	18	1	2	0	3	2	3	2		2	2	2	4	21
			11.1	64.5	81.8	4.7	10		10.3	10.0	17.6	11.1		9.0	15.38	10.0	15.3	29.1
Blood	0	0	5	1	2	9	9	2	7	7	3	6		7	4	9	7	18
			27.7	3.2	9.0	42.8	45.0	28.5	24.1	35.0	17.6	33.3		31.8	30.7	45.0	26.9	25.0
Body fluids	0	1	1	1	0	0	0	0	3	1	1	1		0	0	0	2	10
		4.7	5.5	3.2					10.3	5	5.8	5.5					7.6	13.8

IMI: imipenem; ERT: ertapenem; PIP: piperacillin; GEN: gentamycin; CIP: ciprofloxacin; AMK: amikacin; FOX: cefoxitin; AZT: aztreonam; OFL: ofloxacin; PIP/TAZ: piperacillin/tazobactam; TMP/SMX: trimethoprim/sulfamethoxazole; CEP: cefepime; MDR: multidrug-resistant; XDR: extensively drug-resistant; AmpC: ampicillins C; MBL: metallo-*β*-lactamase.

## Data Availability

The data that support the findings of this study are available from the corresponding author upon reasonable request.

## References

[1] Yadav S. K., Bhujel R., Hamal P., Mishra S. K., Sharma S., Sherchand J. B. (2020). Burden of multidrug-resistant acinetobacter baumannii infection in hospitalized patients in a tertiary care hospital of Nepal. *Infection and Drug Resistance*.

[2] Liu C., Chang Y., Xu Y. (2018). Distribution of virulence-associated genes and antimicrobial susceptibility in clinical *Acinetobacter baumannii* isolates. *Oncotarget*.

[3] Abdi H., Hormozi B., Najimi M. High prevalence of iron acquisition genes among *Acinetobacter baumannii* strains isolated from patients with urinary tract infections in southeast of Iran. *Avicenna Journal of Clinical Microbiology and Infection*.

[4] Karah N., Khalid F., Wai S. N. (2020). Molecular epidemiology and antimicrobial resistance features of *Acinetobacter baumannii* clinical isolates from Pakistan. *Annals of Clinical Microbiology and Antimicrobials*.

[5] Ayobami O., Willrich N., Harder T., Okeke I. N., Eckmanns T., Markwart R. (2019). The incidence and prevalence of hospital-acquired (carbapenem-resistant) *Acinetobacter baumannii* in Europe, Eastern Mediterranean and Africa: a systematic review and meta-analysis. *Emerging Microbes & Infections*.

[6] Dehbashi S., Tahmasebi H., Arabestani M. R. (2018). Association between beta-lactam antibiotic resistance and virulence factors in AmpC producing clinical strains of *P. aeruginosa*. *Osong Public Health and Research Perspectives*.

[7] Tahmasebi H., Maleki F., Dehbashi S. (2019). Role and function of KPC and MBL enzymes in increasing the pathogenicity of pseudomonas aeruginosa isolated from burn wounds. *Journal of Babol University of Medical Sciences*.

[8] Abd El-Baky R. M., Farhan S. M., Ibrahim R. A., Mahran K. M., Hetta H. F. (2020). Antimicrobial resistance pattern and molecular epidemiology of ESBL and MBL producing *Acinetobacter baumannii* isolated from hospitals in Minia, Egypt. *Alexandria Journal of Medicine*.

[9] Tahmasebi H., Dehbashi S., Arabestani M. R. (2020). Co-harboring of mcr-1 and *β*-lactamase genes in *Pseudomonas aeruginosa* by high-resolution melting curve analysis (HRMA): molecular typing of superbug strains in bloodstream infections (BSI). *Infection, Genetics and Evolution*.

[10] Avila-Novoa M.-G., Solís-Velázquez O.-A., Rangel-López D.-E., González-Gómez J.-P., Guerrero-Medina P.-J., Gutiérrez-Lomelí M. (2019). biofilm formation and detection of fluoroquinolone- and carbapenem-resistant genes in multidrug-resistant *Acinetobacter baumannii*. *Canadian Journal of Infectious Diseases and Medical Microbiology*.

[11] Anane A. Y., Apalata T., Vasaikar S. (2019). Prevalence and molecular analysis of multidrug-resistant *Acinetobacter baumannii* in the extra-hospital environment in Mthatha, South Africa. *Brazilian Journal of Infectious Diseases*.

[12] Page M. G. P. (2019). The role of iron and siderophores in infection, and the development of siderophore antibiotics. *Clinical Infectious Diseases*.

[13] Aghajani Z., Rasooli I., Mousavi Gargari S. L. (2019). Exploitation of two siderophore receptors, BauA and BfnH, for protection against *Acinetobacter baumannii* infection. *APMIS*.

[14] Penwell W. F., Arivett B. A., Actis L. A. (2012). The *Acinetobacter baumannii* entA gene located outside the acinetobactin cluster is critical for siderophore production, iron acquisition and virulence. *PLoS One*.

[15] Dorsey C. W., Beglin M. S., Actis L. A. (2003). Detection and analysis of iron uptake components expressed by *Acinetobacter baumannii* clinical isolates. *Journal of Clinical Microbiology*.

[16] CLSI (2019). *Performance Standards for Antimicrobial Susceptibility Testing: 29nd Informational Supplement CLSI M100-S29*.

[17] Tahmasebi H., Dehbashi S., Jahantigh M., Arabestani M. R. (2020). Relationship between biofilm gene expression with antimicrobial resistance pattern and clinical specimen type based on sequence types (STs) of methicillin-resistant *S. aureus*. *Molecular Biology Reports*.

[18] Lukovic B., Gajic I., Dimkic I. (2020). The first nationwide multicenter study of *Acinetobacter baumannii* recovered in Serbia: emergence of OXA-72, OXA-23 and NDM-1-producing isolates. *Antimicrobial Resistance and Infection Control*.

[19] Farajzadeh Sheikh A., Savari M., Abbasi Montazeri E., Khoshnood S. (2020). Genotyping and molecular characterization of clinical *Acinetobacter baumannii* isolates from a single hospital in Southwestern Iran. *Pathogens and Global Health*.

[20] Kamali M., Manshouri S., Bagheri Y. (2020). Prevalence and antibiotic resistance of *Acinetobacter baumannii* among patients in postcardiac surgery intensive care units of Rajaei Hospital, Tehran. *Medical Journal of the Islamic Republic of Iran*.

[21] Armin S., Karimi A., Fallah F. (2015). Antimicrobial resistance patterns of *Acinetobacter baumannii*, *Pseudomonas aeruginosa* and *Staphylococcus aureus* isolated from patients with nosocomial infections admitted to Tehran hospitals. *Archives of Pediatric Infectious Diseases*.

[22] Jasemi S., Douraghi M., Adibhesami H. (2016). Trend of extensively drug-resistant *Acinetobacter baumannii* and the remaining therapeutic options: a multicenter study in Tehran, Iran over a 3-year period. *Letters in Applied Microbiology*.

[23] De Francesco M. A., Ravizzola G., Peroni L., Bonfanti C., Manca N. (2013). Prevalence of multidrug-resistant *Acinetobacter baumannii* and *Pseudomonas aeruginosa* in an Italian hospital. *Journal of Infection and Public Health*.

[24] Banerjee T., Mishra A., Das A. (2018). High prevalence and endemicity of multidrug resistant *Acinetobacter spp.* in intensive care unit of a tertiary care hospital, Varanasi, India. *Journal of Pathogens*.

[25] Kaur A., Singh S. (2018). Prevalence of extended spectrum betalactamase (ESBL) and metallobetalactamase (MBL) producing *Pseudomonas aeruginosa* and *Acinetobacter baumannii* isolated from various clinical samples. *Journal of Pathogens*.

[26] Ain N. U., Iftikhar A., Bukhari S. S. (2018). High frequency and molecular epidemiology of metallo-*β*-lactamase-producing gram-negative bacilli in a tertiary care hospital in Lahore, Pakistan. *Antimicrobial Resistance and Infection Control*.

[27] Batra P., Khurana S., Govindaswamy A. (2019). Antibiotic resistance profile and co-production of extended spectrum beta lactamases and AmpC in Acinetobacter spp. in a level 1 trauma center from India. *Journal of Laboratory Physicians*.

[28] Zeighami H., Valadkhani F., Shapouri R. (2019). Virulence characteristics of multidrug resistant biofilm forming *Acinetobacter baumannii* isolated from intensive care unit patients. *BMC Infectious Diseases*.

[29] Kröger C., Kary S. C., Schauer K. (2017). Genetic regulation of virulence and antibiotic resistance in *Acinetobacter baumannii*. *Genes*.

[30] Song W. Y., Jeong D., Kim J., Lee M. W., Oh M. H., Kim H. J. (2017). Key structural elements for cellular uptake of acinetobactin, a major siderophore of *Acinetobacter baumannii*. *Organic Letters*.

[31] Ribeiro M., Simões M. (2019). Advances in the antimicrobial and therapeutic potential of siderophores. *Environmental Chemistry Letters*.

